# Maternal near miss and death among women with severe hypertensive disorders: a Brazilian multicenter surveillance study

**DOI:** 10.1186/1742-4755-11-4

**Published:** 2014-01-16

**Authors:** Elvira Zanette, Mary Angela Parpinelli, Fernanda Garanhani Surita, Maria Laura Costa, Samira Maerrawi Haddad, Maria Helena Sousa, Joao Luiz Pinto e Silva, Joao Paulo Souza, Jose Guilherme Cecatti

**Affiliations:** 1 Department of Obstetrics and Gynecology, University of Campinas (UNICAMP), School of Medical Sciences, R. Alexander Fleming, 101, P. O. Box 6030, 13083-881 Campinas, São Paulo, Brazil; 2 Centre for Research on Reproductive Health of Campinas (Cemicamp), Campinas, São Paulo, Brazil

**Keywords:** Organ dysfunction, Organ failure, Severe preeclampsia, Eclampsia, Maternal mortality, Maternal morbidity

## Abstract

**Background:**

Hypertensive disorders represent the major cause of maternal morbidity in middle income countries. The main objective of this study was to identify the prevalence and factors associated with severe maternal outcomes in women with severe hypertensive disorders.

**Methods:**

This was a cross-sectional, multicenter study, including 6706 women with severe hypertensive disorder from 27 maternity hospitals in Brazil. A prospective surveillance of severe maternal morbidity with data collected from medical charts and entered into *OpenClinica®*, an online system, over a one-year period (2009 to 2010). Women with severe preeclampsia, severe hypertension, eclampsia and HELLP syndrome were included in the study. They were grouped according to outcome in near miss, maternal death and potentially life-threatening condition. Prevalence ratios and 95% confidence intervals adjusted for cluster effect for maternal and perinatal variables and delays in receiving obstetric care were calculated as risk estimates of maternal complications having a severe maternal outcome (near miss or death). Poisson multiple regression analysis was also performed.

**Results:**

Severe hypertensive disorders were the main cause of severe maternal morbidity (6706/9555); the prevalence of near miss was 4.2 cases per 1000 live births, there were 8.3 cases of Near Miss to 1 Maternal Death and the mortality index was 10.7% (case fatality). Early onset of the disease and postpartum hemorrhage were independent variables associated with severe maternal outcomes, in addition to acute pulmonary edema, previous heart disease and delays in receiving secondary and tertiary care.

**Conclusions:**

In women with severe hypertensive disorders, the current study identified situations independently associated with a severe maternal outcome, which could be modified by interventions in obstetric care and in the healthcare system. Furthermore, the study showed the feasibility of a hospital system for surveillance of severe maternal morbidity.

## Background

It has been estimated that hypertensive disorders (HD) in pregnancy cause 50.000 maternal deaths (MD) annually in the world and the vast majority of them occur in low-income or middle-income countries [1]. The main cause of MD in Latin America and in the Caribbean is HD, accounting for approximately one-fourth of the total number of deaths [2].

In high-income countries, such as the United States, the prevalence of hospital admissions due to HD in pregnancy increased significantly between 1998 and 2006, rising from 67.2 to 81.4 per 1.000 deliveries over that time period [3]. In Canada, HD is the third cause of antenatal hospitalization, with little variation in rates for a 10-year period, from 15.4 per 1000 deliveries in 1991 to 11.3 per 1000 deliveries in 2001 [4].

HD increase the risk of severe complications by 3 to 25 times, e.g. placental abruption, thrombocytopenia, disseminated intravascular coagulation, acute pulmonary edema, cerebrovascular disorders and other conditions, in comparison to women without hypertension [3,5,6]. The contrast between low or very low maternal mortality ratios (MMR) in high-income countries, compared to low-income or middle-income countries with high MMR has been attributed to the quality of obstetric care, patient access to hospitalization, qualification of health professionals and structural resources, including the input and availability of intensive care units [7-9].

In this context, the United Nations has recognized that quality of care is of central importance in the strategy to improve maternal and infant health [10]. In the last two decades, there has been increasing interest in the study of severe maternal morbidity (SMM) cases, including its more severe component - the maternal near miss (NM), as a supplementary method to audits and inquiries on MD [11-13]. The investigation of NM could furnish more details about factors contributing to both mortality and SMM [10-14] and may be a reference for quality assessment of obstetric care. Nevertheless, in the literature there is still a wide variety of criteria used to identify maternal NM cases [14].

In 2009, the WHO defined concepts and standardized criteria to identify NM cases, after the identification of organ dysfunction and/or failure as the main determinants of severity. Clinical signs, laboratory tests and management interventions were used, all capable of diagnosing organ dysfunction or failure [15]. These criteria were previously validated by the WHO Working Group following markers of dysfunction and total maximum SOFA (sequential organ failure assessment) score, applied to an obstetric population [15-17].

This study is proposed due to the high association between hypertensive disorders in pregnancy and severe obstetric/clinical complications, resulting in high rates of severe maternal outcomes (SMO). In addition, there are only few studies to date focused on severe morbidity as proposed by the WHO [15]. Therefore, the purpose of the current study was to identify the prevalence and factors associated with the risk of severe maternal outcomes (NM and MD) in a female population with severe hypertensive disorders (severe preeclampsia, eclampsia, severe hypertension and HELLP syndrome) [18].

## Method

### Study population

This was a multicenter study, based on 27 referral maternity hospitals located in the five Brazilian regions participating in the Brazilian Network for Surveillance of Severe Maternal Morbidity [19]. Local investigators from each participating maternity hospital carried out a prospective surveillance with data routinely collected, but only after discharge (or death) in all women admitted to hospital during the study period because of a SMM episode. We defined a SMM condition as any case that could be classified, according to the WHO recommendations [15], as a Potentially Life Threatening condition or a severe maternal outcome (Near Miss or Maternal Death). Data were first collected in specific forms and then entered into the online OpenClinica® platform. Details on the original study methods are already published elsewhere [19,20].

### Identification of cases

This is an analysis of SMM cases due to severe hypertensive disorders among the total number of SMM during pregnancy and postpartum period, managed by these maternity hospitals, over a 12-month period, from July 2009 to June 2010. For this specific analysis focusing on severe hypertensive disorders, we included women diagnosed with: severe hypertension and hypertensive emergency: blood pressure (BP) level ≥ 160/110 mmHg or hypertensive peak of any value, associated with symptoms or signs of target organ lesion; severe preeclampsia: BP ≥ 160/110 mmHg and/or symptomatology of target organ compromise and/or proteinuria determined by dipstick ++ or over 24 hours ≥ 2 g; and/or oliguria < 30 ml/hour and/or thrombocytopenia < 100.000 mm3; eclampsia: presence of seizures in a woman with preeclampsia; and HELLP syndrome: presence of at least one parameter: LDH ≥600U/L; bilirubin ≥1.2 mg/dl; AST ≥70U/L; or thrombocytopenia < 100.000 mm^3^[18,20]. ^.^The cases were classified according to the WHO criteria into PLTC and severe maternal outcome (NM/MD) [15]. All these definitions and criteria were included in the correspondent manual of operation of the study [20].

### Variables

The variables studied were: socio demographic characteristics, obstetric history, history of previous disease, patient access to obstetric care, mode of delivery, perinatal results, clinical complications and advanced life support interventions (excluding all those already defined as near miss criteria [15]), in addition to identification of the “three delays” according to the model originally developed by Taddeus and Maine [21]. The questionnaire has a specific session where delays are explored separately for each of the three phases, with several possibilities for each one being directly asked. Besides that, a rigorous system of data checking was developed by the coordinating center in order to gather more reliable information, as already described [20]. The adequacy of the number of prenatal care visits according to gestational age was based on the Brazilian Ministry of Health recommendation that at least one visit should be performed during the first trimester, two during the second and three during the third trimester of pregnancy. The variables were selected, among all others available in the database, after some exploratory analysis and because they were supposed to be the most important ones for the topic of care to women with severe hypertensive disorders during pregnancy.

### Analysis

Initially, the health indicators for severe maternal morbidity, namely Maternal Near Miss Ratio (MNMR), Severe Maternal Outcome Ratio (SMOR), Maternal Near Miss to Maternal Death Ratio (NM:MD ratio), Mortality Index (a proxy to case fatality ratio) and Maternal Mortality Ratio (MMR) were calculated as recommended by WHO [15]. Bivariate analysis was then performed to identify factors (predictors) associated with severe maternal outcomes (maternal near miss or maternal death) by estimating prevalence ratios (PR) and their respective 95% confidence intervals (CI), adjusted for cluster effect (maternity hospitals or centers), although the intraclass correlation coefficients were very low for the majority of variables studied as desired and already described [22]. Maternal complications and procedures used for management other than those already used for near miss case definition according to WHO were described comparatively among women from both groups, with differences assessed with Chi-square test. Additionally the risks of perinatal results were also estimated for women who had severe maternal outcomes (near miss or death) with adjusted prevalence ratios (PR) and their respective 95% confidence intervals (CI). Finally, Poisson multiple regression analysis was performed, also adjusted by cluster and all other predictors. A 95% confidence level was used (with a 5% level of significance) and the software used for analyses were SPSS® version 17 (SPSS, Chicago, IL, USA) and Stata® version 7.0 (StataCorp, College Station, TX, USA).

### Ethics statement

The study was approved by each local Institutional Review Board and by the National Human Research Ethics Committee, under the letter of approval 097/2009.

## Results

In the one-year study period, there were 82,144 live births in the 27 maternity hospitals participating in the study and 9555 women received a diagnosis of SMM. Severe hypertensive disorders were associated with 70% of these hospital admissions (6706/9555), corresponding to 81.6 cases per 1000 deliveries. Among the total number of women with severe hypertensive disorders, 94% were classified as PLTC (6315/6706), while 349 cases had organ dysfunction or failure, with prevalence of 4.2 NM cases per 1000 LB, a mortality index of 10.7% (42 MD per 391 cases of NM plus MD), and the maternal near miss to maternal death ratio was 8.3 NM cases to 1 MD (Table 1). The mortality was almost half of that for non-hypertensive disorder conditions.

**Table 1:** Women with hypertensive disorders (HD) and non HD among those with severe maternal morbidity or death and their correspondent health indicators

**Condition**	**PLTC**	**NM**	**MD**	**Total**
	**n (%)**	**n (%)**	**n (%)**	**N (%)**
HD	6315 (73)	349 (45)	42 (30)	6706 (70)
Non HD	2330 (27)	421 (55)	98 (70)	2849 (30)
Total	8645 (100)	770 (100)	140 (100)	9555 (100)

PLTC: Potentially life-threatening condition; NM: near miss; MD: maternal death; Live births = 82,144.

MNM prevalence ratio for HD = 4.2 NM/1000LB (for non HD = 5.1 NM/1000LB).

Severe Maternal Outcome Ratio (SMOR) for HD = 4.76/1000LB (for non HD = 6.32/1000LB).

Maternal near miss: Maternal death ratio for HD = 8.3NM:1MD (for non HD = 4.3NM:1MD).

Mortality index for HD = 42MD/391 = 10.7% (for non HD = 18.9%).

MMR = 51.1MD/100.000 LB for HD (for non HD = 119.3 MD/100.000LB).

Table 2 highlights the percentage of approximately 20% of adolescents with SMM due to severe hypertensive disorders, without any significant association with severe maternal outcome. Age older than 40 years increased the risk of SMO by almost 70% (PR 1.67; IC 1.21 – 2.31), which was not confirmed by multivariate regression analysis. The risk of SMO decreased about 40% in women self-reported as non-white and in those without a steady partner on hospital admission, but these findings were not confirmed in the multivariate analysis. Maternal history of some chronic diseases such as kidney disease (PR 4.17; 2.49 – 6.98), connective tissue disorders (PR 4.30; 2.16 – 8.56) and heart diseases (PR 4.09; 2.18 – 7.64) increased around fourfold the risk of SMO. The prevalence of chronic arterial hypertension was 22.9% and of diabetes was 2.7% in the total number of women with severe HD without significant association with the outcome. History of heart disease was independently associated with a severe maternal outcome by regression analysis (PR_adj_ 1.98; 1.19 – 3.29).

**Table 2:** **Estimated risk of severe maternal outcome (SMO** = **NM + MD) among women with severe hypertensive disorders during pregnancy or childbirth according to maternal characteristics, obstetric and medical history**

**Predictors**	**%**	**SMO**		**PLTC**		**PR**	**95% CI**
	**Total**	**n**	**%**	**n**	**%**		
**Age (years)**							
≤ 19	18.7	65	16.6	1186	18.8	0.98	0.77 – 1.25
20 – 29	46.9	167	42.7	2976	47.1	(ref)	
30 – 39	29.6	130	33.2	1856	29.4	1.23	0.98 – 1.55
≥ 40	4.9	29	7.4	297	4.7	**1.67**	**1.21 – 2.31**
**Skin color (a)**							
White	36.3	136	46.4	1623	35.7	(ref)	
Non white	63.7	157	53.6	2927	64.3	**0.66**	**0.48 – 0.91**
**Education (b)**							
Elementary	46.0	128	51.2	2162	45.7	1.23	0.88 – 1.73
> elementary	54.0	122	48.8	2566	54.3	(ref)	
**Marital status (c)**							
With partner	52.7	207	67.6	2783	51.8	(ref)	
Without	47.3	99	32.4	2589	48.2	**0.53**	**0.37 – 0.76**
**Number of pregnancies (d)**							
1	45.7	159	41.4	2893	45.9	0.91	0.72 – 1.16
2 – 3	37.0	141	36.7	2330	37.0	(ref)	
4 or more	17.4	84	21.9	1076	17.1	1.27	1.00 – 1.61
**Number of Childbirths (d)**							
0	52.4	178	46.4	3327	52.8	0.80	0.65 – 1.00
1 – 2	36.3	153	39.8	2270	36.0	(ref)	
3 or more	11.3	53	13.8	702	11.1	1.11	0.76 – 1.63
**Previous C-sections (e)**							
0	76.8	282	75.0	4797	76.9	(ref)	
1 or more	23.2	94	25.0	1442	23.1	1.10	0.86 – 1.41
**Medical history (f)**							
Chronic hypertension	22.9	92	28.0	1243	22,6	1.30	1.00 – 1.70
Diabetes	2.7	16	4.9	139	2.5	1.87	1.00 – 3.49
Kidney disease	1.3	17	5.2	58	1.1	**4.17**	**2.49 – 6.98**
Collagen disorders	0.4	6	1.8	19	0.3	**4.30**	**2.16 – 8.56**
Heart disease	1.5	19	5.8	67	1.2	**4.09**	**2.18 – 7.64**
Smoking	3.5	12	3.6	194	3.5	1.03	0.50 – 2.11
**Total**		391		6315			

PR: prevalence ratio adjusted by cluster effect.

Missing data: (a) 1863 cases; (b) 1728 cases; (c) 1028 cases; (d) 23 cases; (e) 91 cases; (f) 888 cases.

Values in bold mean they are statistically significant.

Table 3 shows that during pregnancy and at the time of hospital admission, healthcare insurance was mainly provided by the Brazilian national public healthcare system and this was not associated with severe maternal outcome. Furthermore, the adequacy of prenatal care, evaluated by the number of visits for each gestational age, was appropriate in more than 70% of the total number of cases, with no association with SMO. Lower gestational age at the time of hospital admission due to severe hypertensive disorders (early onset of disease) and also postpartum admission were strongly associated with severe maternal outcome. In addition, women with SMO had about twice the rate of elective Caesarean sections. Emergency access to an obstetric referral center by ambulance, even in a scheduled transference was two to three times higher in the SMO group; the diagnosis of NM already existed in around 30% of cases at the time of patient admission to study maternity hospitals (data not shown in table). Most women with severe hypertensive disorders suffered some kind of delay in receiving care (55.6%) and these went to the second type, i.e. related to the healthcare system (PR 2.86; 1.89 – 4.33) and third type, those related to healthcare professionals (PR 2.45; 1.53 – 3.92) (Table 4).

**Table 3:** **Estimated risk of severe maternal outcome (SMO** = **NM + MD) among women with severe hypertensive disorders during pregnancy or childbirth according to characteristics of current pregnancy and delays in obstetric care**

**Predictors**	**Total**	**SMO**		**PLTC**		**PR**	**95% CI**
	**%**	**n**	**%**	**n**	**%**		
**Prenatal coverage** (a)							
Public	92.7	291	90.7	4566	92.6	0.76	0.47 – 1.23
Other	7.3	30	9.3	351	7.1	(ref)	
**Prenatal adequacy** (b)							
No	23.6	84	21.9	1486	23.7	0.91	0.71 – 1.15
Yes	76.4	299	78.1	4771	76.3	(ref)	
**Hospitalization coverage** (c)							
Public	99.3	386	98.7	6269	99.4	0.53	0.17 – 1.66
Other	0.7	5	1.3	41	0.6	(ref)	
**Gestational age at hospital admission** (d)							
<22	1,5	12	3.1	86	1.4	**5.06**	**2.60 – 9.82**
22 – 27	4.9	35		288	4.6	**4.47**	**2.53 – 7.92**
28 – 33	20.7	111	28.9	1265	20.2	**3.33**	**2.21 – 5.02**
34 – 36	22.5	89	23.2	1409	22.5	**2.45**	**1.59 – 3.79**
≥37	47.2	76	19.8	3062	48.9	(ref)	
Postpartum	3.2	61	15.9	152	2.4	**11.82**	**7.59 – 18.43**
**Gestational age at delivery** (e)							
<22	0.5	3	0.9	30	0.5	**3.23**	**1.13 – 9.28**
22 – 27	3.0	27	7.8	172	2.8	**4.83**	**2.72 – 8.57**
28 – 33	17.5	110	31.8	1030	16.7	**3.43**	**2.19 – 5.38**
34 – 36	22.6	96	27.7	1380	22.3	**2.31**	**1.57 – 3.41**
≥37	51.8	95	27.5	3284	53.1	(ref)	
Still pregnant	4.6	15	4.3	284	4.6	**1.78**	**1.03 – 3.10**
**Mode of delivery** (f)							
C-section	74.8	309	79.8	4694	74.5	1.33	0.99 – 1.79
Other	25.2	78	20.2	1605	25.5	(ref)	
**Onset of labor** (g)							
Spontaneous	26.8	66	17.7	1716	27.4	(ref)	
Induction	9.0	33	8.9	562	9.0	1.50	0.86 – 2.60
Elective C-section	59.0	254	68.3	3665	58.5	**1.75**	**1.11 – 2.76**
Abortion	0.6	4	1.1	37	0.6	**2.63**	**1.05 – 6.59**
Still pregnant	4.5	15	4.0	285	4.5	1.35	0.77 – 2.37
**Access to referral center** (h)							
Not scheduled	10.2	69	18.4	576	9.7	**2.98**	**1.62 – 5.47**
Scheduled	46.4	207	55.3	2715	45.9	**1.97**	**1.30 -2.97**
Spontaneous	43.3	98	26.2	2628	44.4	(ref)	
**Delay**s							
Women/family members (i)	39.9	146	44.9	2179	39.6	1.23	0.89 – 1.69
Health service (j)	16.2	129	35.6	874	15.0	**2.86**	**1.89 – 4.33**
Health professional (l)	19.1	132	36.6	1066	18.0	**2.45**	**1.53 – 3.92**
Any delays	55.6	256	73.6	3131	54.5	**2.22**	**1.44 – 3.43**
**Total**		391	100	6315	100		

Values in bold mean they are statistically significant; PR = prevalence ratio adjusted by cluster effect.

Missing data: (a) 1468 cases; (b) 66 cases; (c) 5 cases; (d) 60 cases; (e) 180 cases; (f) 20 cases; (g) 69 cases; (h) 413; (i) 875 cases; (j) 513 cases; (l) 613 cases.

**Table 4:** **Other maternal complications and estimated risks of perinatal results of current pregnancy associated with severe maternal outcomes (SMO** = **NM + MD) among women with severe hypertensive disorders during pregnancy or childbirth**

**Variables**	**%**	**SMO**		**PLTC**		**p**	
	**Total**	**n**	**%**	**n**	**%**		
Maternal complication*							
Placental abruption	3.6	37	9.5	207	3.3	<0.0001	
Postpartum hemorrhage	4.0	74	18.9	196	3.1	<0.0001	
Other severe hemorrhage	0.5	17	4.3	19	0.3	<0.0001	
Pulmonary edema	1.7	65	16.6	48	0.8	<0.0001	
Thromboembolism	0.2	2	0.5	9	0.1	0.2688	
Sepsis	1.0	49	12.5	15	0.2	<0.0001	
Procedures used for management*	79.4	384	98.2	4938	78.2	<0.0001	
Blood transfusion	7.9	207	52.9	326	5.2	<0.0001	
ICU admission	21.9	290	74.2	1178	18.7	<0.0001	
Hospital stay >7days	30.7	253	64.7	1805	28.6	<0.0001	
Invasive mechanical ventilation	1.8	116	29.7	2	0.0	<0.0001	
Magnesium sulphate use	68.4	279	71.4	4309	68.2	0.2178	
**Perinatal results**						**PR**	**95% CI**
Apgar at 5 min <7 (a)	3.3	34	12.9	164	2.9	**4.28**	**3.26 – 7.38**
Apgar at 5 min ≥7	96.7	230	87.1	5505	97.1	(ref)	
Birth weight <2500 g (b)	42.3	212	68.6	2388	40.9	**2.98**	**2.10 – 4.22**
Birth weight ≥2500 g	57.7	97	31.4	3447	59.1	(ref)	
Stillbirth (c)	4.2	60	17.7	203	3.4	**4.90**	**3.26 – 7.38**
Live birth	95.8	279	82.3	5715	96.6	(ref)	
Neonatal death (d)	2.4	11	4.2	124	2.3	**2.50**	**1.35 – 4.60**
NICU admission (d)	22.3	107	41.3	1172	21.4	**2.56**	**1.53 – 4.28**
Hospital discharge (d)	75.3	141	54.4	4179	76.3	(ref)	
**Total**		391	100	6315	100		

*excluded all near miss criteria according to WHO; risks are not estimated because they are not predictors nor outcomes.

p-values reported derive from Chi-square tests.

PR: prevalence ratio adjusted by cluster effect.

Missing data: (a) = 773 cases; (b) = 562 cases; (c) = 449 cases; (d) = 972 cases.

Values in bold mean they are statistically significant.

Table 4 shows that other maternal complications (unless thromboembolism) and procedures used for the management of complications (unless the use of magnesium sulphate), other than those already used for maternal near miss case definition according to WHO [15], are significantly more prevalent among cases of SMO than among cases of PLTC as they were obviously expected. Magnesium sulphate was used in a little more than two thirds of the total number of cases (68.4%) and showed no significant difference among groups. The risks of worse perinatal results were higher, especially among SMO cases, with 13% of hypoxic newborns, almost 70% with low birth weight and more than 40% were admitted to NICU. Perinatal mortality was 6.6%, with 21.9% versus 5.7% among women with SMO compared to those with PLTC, respectively (the sum of stillbirths plus neonatal deaths, not showed in table).

In Table 5, Poisson multiple regression analysis identified variables independently associated with severe maternal outcomes cases: history of heart disease, acute pulmonary edema, postpartum hemorrhage and early onset of disease in relation to gestational age, or hypertension remote from term. Blood product transfusion, admission to ICU, invasive mechanical ventilation non-related to anesthesia and longer periods of hospitalization were highlighted as significant management procedures associated with worse outcome. Delays in obtaining adequate obstetric care were also identified by difficulties linked to healthcare services and health professionals.

**Table 5:** **Poisson multiple regression analyses with variables independently associated with severe maternal outcome (SMO** = **NM + MD; N** = 5488*) among women with severe hypertensive disorders during pregnancy or childbirth

**Variables**	**PR**_**adj**_	**95% CI**	**p**
**Clinical**			
Gestational age <37 weeks or postpartum at hospital admission	1.73	1.22 – 2.44	0.003
Postpartum hemorrhage	1.60	1.28 – 2.00	<0.001
Pulmonary edema	1.86	1.24 – 2.82	0.005
Previous heart disease	1.98	1.19 – 1.59	0.029
**Management**			
Blood transfusion	3.67	2.48 – 5.44	<0.001
Invasive mechanical ventilation	2.83	1.99 – 4.01	<0.001
ICU admission	3.75	2.06 – 6.82	<0.001
Hospital stay >7days	1.66	1.18 – 2.33	0.005
**Delays**			
Delay: health system	1.60	1.19 – 3.29	0.010
Delay: health professional	1.28	1.03 – 1.59	0.029

PR_adj_ = prevalence ratio adjusted by cluster effect and all other predictors entering the model of Poisson multivariate regression analysis (maternal age, skin color, education, marital status, number of pregnancies, number of childbirths, previous C-sections, history of medical conditions, smoking, prenatal coverage, prenatal adequacy, hospitalization coverage, gestational age at hospital admission, gestational age at delivery, mode of delivery, onset of labor, access to referral center, and delays).

*There were 1218 cases excluded due to at least one missing value for variables entering the model

## Discussion

In this study, we applied the Near Miss criteria according to WHO recommendation [15] to a group of women with severe hypertensive disorders, following clinical diagnoses of severe preeclampsia, eclampsia, HELLP syndrome and severe arterial hypertension/hypertensive emergency, justified by a greater specificity of these criteria to case severity. Therefore, the capacity to identify clinical, obstetric and/or epidemiological markers for organ dysfunction and/or failure was increased in this group of women. We also performed the analysis of delays after a systematic evaluation of data collected for interventions that are beneficial to a reduction in morbidity and mortality due to hypertension [23,24], such as the use of magnesium sulphate and the collection of variables related to obstetric care, such as patient access to referral centers, among others. None of these results are “not already known”, however the confirmation of these associations in a large number of cases prospectively followed is important in establishing firm health care surveillance policies, be it in hospitals, and especially if to be government related, wherever correspondent policies are being planned.

To increase the likelihood of identifying SMO markers in this group of women, we conducted a multicenter study in 27 referral maternity hospitals. Most of these hospitals were public and rendered service by the Brazilian national healthcare system. These maternity hospitals were distributed in the five Brazilian regions and had at least 1000 deliveries per year. With a prospective surveillance system for identifying cases of severe maternal morbidity admitted to hospital, it was possible to collect data on all women and select those hospitalized due to severe hypertensive disorder for this study. Our results showed a rate of 81.6 SMM cases of severe hypertensive disorders per 1000 LB. These numbers create demand for hospital beds specialized in the management of these conditions and corroborate the high prevalence of complications that result in hospitalizations due to hypertensive disorders in general [2,3].

With this method, we identified a prevalence of 4.2 NM cases due to severe hypertensive disorders per 1000 LB and a ratio of 8.3 cases of NM to 1 death, and mortality index of 10.7%. This was much higher than those found in high-income countries, which was 0 to 1.8% [25-27] and in middle and low-income countries (0.5 to 20.7%) [12,28-31]. It is noteworthy that studies in high-income countries used mainly clinical criteria for the diagnosis of NM (severe preeclampsia, eclampsia and HELLP syndrome). Studies of low-income countries selected cases of organ dysfunction and/or failure among clinical diagnoses and therefore used a method similar to ours. In this study, a high ratio of specific maternal mortality associated with hypertensive disorders was found, with 51 deaths per 100.000 LB in these maternity hospitals.

Among the maternal socio demographic, obstetric, and clinical variables, older maternal age was identified as a risk factor for severe maternal outcome, a finding that is in agreement with results in the literature [32]. Some comorbid conditions that are relevant in studies investigating severe maternal morbidity are also associated with a severe maternal outcome, not only for the hypertensive disorder group [32]. Contrary to what has been reported in the literature, non-white color, marital status and living with a steady partner were variables associated with a lower risk of SMO. Although the same findings were not confirmed in the multivariate analysis, these results were quite surprising. Anyway there are some arguments that could possibly explain them: in Brazil, racial miscegenation is high and race was dichotomously categorized as white or non-white, which may have contributed to reduce differences between groups. Regarding marital status however we found no answers and only identified in the literature a qualitative study conducted in a low-income country reporting a negative influence of partner and family members on the woman’s decision to seek medical care when she perceived symptoms [33]. Another possible explanation for these findings could be a bias due to the relatively high proportion of missing values for these variables. This occurred because the data was gathered after discharge of the women and this information was not in their clinical records. However we did not believe this is very likely to be true, taking into account that a higher proportion of missing values comes from less severe (PLTC) than from NM or MD cases. On multivariate analysis, these variables did not show to be associated with severe outcome. It is also important to consider that all women included in the study already had a severe clinical picture, and this condition may have contributed to a greater homogeneity between groups.

Among the obstetric variables, the most noteworthy is early onset of hypertensive disease, evaluated by the lowest gestational age at the time of admission. This condition is recognized in the specific literature as a predictor of poor maternal and perinatal prognosis [18,34,35]. There is also an increased potential for the presence of subclinical disease, such as systemic lupus erythematous, kidney or thrombotic disease [35-38]. This condition remained an independent variable for severe maternal outcome by multivariate analysis and was confirmed as a marker of poor maternal and perinatal prognosis, reinforcing recommendations of early admission to tertiary or referral centers, for adequate management of the woman and fetus. In addition, assessment of the best time for therapeutic delivery is warranted, since it also results in a higher prevalence of preterm birth and thus increased need for neonatal intensive care therapy. In this study, preterm occurred in more than 40% of the total number of newborn infants and the prevalence was higher in the SMO group. This poor perinatal outcome was in agreement with a population-based study on the impact of hypertensive disorders in perinatal mortality [39].

Among the clinical maternal variables identified by Poisson multiple regression analysis, a history of heart disease remained significantly associated with a severe maternal prognosis. Studies conducted with pulmonary artery catheterization showed that women suffering from chronic arterial hypertension with superimposed preeclampsia had a higher risk of developing pulmonary edema [40] and also heart failure. Sibai suggested that women with chronic arterial hypertension be evaluated in the preconception period or at the beginning of pregnancy for the presence of target organ (heart, kidneys) damage. These cases are termed “high-risk” and prenatal care should be specialized to minimize maternal/perinatal morbidity and mortality [41]. Chronic hypertension was the major comorbid condition identified in around one-fourth of the cases with severe hypertensive disorders.

Acute pulmonary edema is also a clinical marker of cardiopulmonary complication. The pathophysiology of preeclampsia favors the occurrence of this condition. Fluid overload imposed on these women may increase the incidence of pulmonary edema, especially during anesthetic procedures and in the postpartum period [42] and that is why fluid restriction is recommended (60 to 125 ml per hour in 24 hours) in the management of severe preeclampsia [43]. In our study, acute pulmonary edema was prevalent in 1.7% of the total number of cases. Of these, it occurred in 16.6% of the SMO group, while in only 0.8% among patients with PLTC. Acute pulmonary edema has already been elected an indicator for quality assessment of obstetric care in this group of women [44].

Regarding obstetric care, it is well-known that severe hypertensive disorders, particularly eclampsia and hypertensive emergency, predispose patients to the occurrence of hemorrhagic or ischemic cerebral events, due to endothelial dysfunction associated with the loss of cerebral blood flow auto regulation that occurs in the presence of eclamptic seizure or an abrupt elevation of blood pressure levels. In the United Kingdom, a study on the incidence of eclampsia and its complications reported a significant decrease in both conditions between 1992 and 2005, after the introduction of magnesium sulphate for the management of preeclampsia and eclampsia, with no maternal death in 2005 [45]. We did not classify the clinical forms of hypertension and therefore we could not evaluate the use of magnesium sulphate in these cases.

Considering obstetric complications, there is a classic association between severe hypertensive disorders and placental abruption. However, there is no distinction in the occurrence of postpartum hemorrhage. In our study this complication was present in 4% of the total number of cases, with 19% in the SMO group and 3% in the group with PLTC. We believe that placental abruption may represent an intermediate variable for the occurrence of postpartum hemorrhage, due to atony or uterine apoplexy, which may also lead to an increased rate of postpartum hysterectomy due to bleeding. However, this affirmation could not be made because the association between cases of placental abruption and uterine atony was not analyzed. Studies have shown that the use of magnesium sulphate in the antepartum care of these cases decreases the occurrence of placental abruption [6,46] and may reduce this morbid condition as well.

The need for interventions to manage severe disease occurred in about 80% of these cases, with a clear predominance in the SMO group. Among them, mechanical ventilation, blood product transfusion, admission in intensive care units and also a longer period of hospitalization were significantly associated with a worse prognosis. These findings are in agreement with other national studies [47-49] and reinforce the need for obstetric intensive care units for patient management.

Some delays for adequate obstetric care occurred in more than half of the cases and in even a larger proportion of SMO cases. Factors related to healthcare system and services (second delay) and health professionals (third delay) were associated with the worst outcomes. These results unveil a fragmented care network, inefficient offices for regulation of obstetric beds and/or lack of hospital beds for the management of complex cases (obstetric intensive care units) and also a model of care that disrespects scientific evidence on what is beneficial for the treatment of hypertension, also evaluated by the use of magnesium sulphate with a prevalence lower than 70% for the total number of severe cases. These findings are in agreement with publications about barriers against the reduction in MD from hypertension, in low or middle-income countries [8,23,44].

This study has some possible limitations. First, it was not a population-based study, although it had representativeness in maternity hospitals in every region of the country. Second, we did not classify the clinical forms of severe hypertension, a fact that could allow us to identify SMO by specific diagnosis, such as eclampsia which is recognized as the major cause of maternal morbidity and mortality in low-income and middle-income countries [1,6,9]. Classification of hypertension could also assess management with magnesium sulphate in specific clinical situations, since we believe that there was a low prevalence of magnesium sulphate use (68%), considering that all cases were severe. This finding was similar to the results of another study on the prediction of adverse outcome in preeclampsia, in which the prevalence of magnesium sulphate use was only 62% [34]. A new international study from WHO has just showed that magnesium sulphate was used in around 85% of cases of eclampsia, therefore arising the discussion on the appropriateness and timing of use that would probably also have a key role in determining the outcomes for women [50]. Third, we failed to explore some relevant NM aspects by patient interview, due to technical impossibility in obtaining a written informed consent term from all women regardless of clinical condition presented at the time of hospital admission. Nevertheless, it was possible to perform analysis of the delays. In addition, and exactly by this same reason, there are some variables like skin color and education where missing data reached relatively high proportions (around 25% of cases), thus possibly favoring the occurrence of bias. However there is no evidence that they were preferably distributed between categories.

On the other hand, the merit of the study is its large number of cases and the prospective surveillance using standardized definitions recently adopted and recommended by WHO. Poisson multiple regression analysis could be performed in about 5500 cases to identify and/or confirm clinical and obstetric markers independently associated with worse maternal outcome, such as hypertension remote from term, postpartum hemorrhage, history of heart disease and pulmonary edema, which may be modified by direct intervention in patient care. Furthermore, unpublished analysis of the delays identified weaknesses in the national healthcare system, since technical, political and administrative interventions may modify this predictor. Finally, we could not locate other study addressing NM as defined by the WHO related to HD. This reveals the possibility of implementing a prospective, hospital-based and national surveillance system for the investigation of NM, particularly in hypertension which is still the leading cause of maternal death in Brazil.

### Ethical details

The research protocol was approved by the Institutional Review Board of the coordinating institution on 5th May 2009 (Document CEP 027/2009).

## Abbreviations

BP: Blood pressure; HD: Hypertensive disorders; ICU: Intensive care unit; LB: Live births; MD: Maternal death; MMR: Maternal mortality ratio; NICU: Neonatal intensive care unit; NM: Near miss; PLTC: Potentially life threatening condition; PR: Prevalence ratio; SMM: Severe maternal morbidity; SMOR: Severe maternal outcome; SMOR: Severe maternal outcome ratio; SOFA: Sequential organ failure assessment; WHO: World health organization.

## Competing interests

The authors declare that there are no competing interests.

**Brazilian Network for the Surveillance of Severe Maternal Morbidity Group:** Rodolfo C Pacagnella, Rodrigo S. Camargo, Vilma Zotareli, Lúcio T. Gurgel, Lale Say, Robert C Pattinson, Marilza V Rudge, Iracema M Calderon, Maria V Bahamondes, Danielly S Santana, Simone P Gonçalves, Eliana M Amaral, Olímpio B Moraes Filho, Simone A Carvalho, Francisco E Feitosa, George N Chaves, Ione R Brum, Gloria C Saint’Ynes, Carlos A Menezes, Patricia N Santos, Everardo M Guanabara, Elson J Almeida Jr, Joaquim L Moreira, Maria R Sousa, Frederico A Peret, Liv B Paula, Luiza E Schmaltz, Cleire Pessoni, Leila Katz, Adriana Bione, Antonio C Barbosa Lima, Edilberto A Rocha Filho, Melania M Amorim, Debora Leite, Ivelyne Radaci, Marilia G Martins, Frederico Barroso, Fernando C Oliveira Jr, Denis J Nascimento, Cláudio S Paiva, Moises D Lima, Djacyr M Freire, Roger D Rohloff, Simone M Rodrigues, Sergio M Costa, Lucia C Pfitscher, Adriana G Luz, Daniela Guimaraes, Gustavo Lobato, Marcos Nakamura-Pereira, Eduardo Cordioli, Alessandra Peterossi, Cynthia D Perez, Jose C Peraçoli, Roberto A Costa, Nelson L Maia Filho, Jacinta P Matias, Silvana M Quintana, Elaine C Moises, Fátima A Lotufo, Luiz E Carvalho, Carla B Andreucci, Márcia M Aquino, Maria H Ohnuma, Rosiane Mattar and Felipe F Campanharo.

## Authors’ contributions

The idea for the study and this specific analytic approach arose in a group discussion among all the authors. The analyses were planned and performed by EZ, MAP, JGC and MHS. The first version of the manuscript was drafted by EAZ and MAP, and then complemented with suggestions of all the others and mainly of FGS, MLC, SMH, JPS and JLPS. All authors contributed to the development of the study protocol and approved the final version of the manuscript.
